# Artificial intelligence-enabled clinical decision support systems in preadmission testing: a scoping review of risk prediction, triage, and perioperative workflows (2020–2025)

**DOI:** 10.1007/s10877-025-01404-w

**Published:** 2026-01-31

**Authors:** Lawrence Willis Chinn, Isabelle Nemeh, Natasha R. Chinn

**Affiliations:** 1 Department of Anesthesiology and Perioperative Medicine, Rutgers New Jersey Medical School, 185 South Orange Avenue, Newark, NJ 07103 USA; 2 Englewood Hospital and Medical Center, 350 Engle Street, Englewood, NJ 07631 USA

**Keywords:** Artificial intelligence, Clinical decision support systems, Machine learning, Preadmission testing, Perioperative assessment, Risk prediction

## Abstract

Preadmission testing (PAT) is a critical step in perioperative care that supports risk stratification, triage, and optimization. Tools such as the American Society of Anesthesiologists Physical Status (ASA-PS) classification have limitations. This review mapped evidence on artificial intelligence–enabled clinical decision support systems (AI-enabled CDSS) and risk prediction tools in PAT and perioperative assessment, with particular attention to their implications for perioperative efficiency and patient safety. A scoping review was conducted following the Preferred Reporting Items for Systematic Reviews and Meta-Analyses extension for Scoping Reviews guidelines. PubMed, Embase, Scopus, and CINAHL were searched for English-language studies published between January 1, 2020, and August 1, 2025. Eligible studies applied artificial intelligence (AI) or machine learning (ML) to preoperative or PAT–related evaluation, risk prediction, triage, or decision support. Two reviewers independently screened all records. The review was preregistered on the Open Science Framework (DOI: 10.17605/OSF.IO/JKCRH). The original registration described a broader “digital determinants of health” scope, which was refined to AI-enabled CDSS before data extraction. Fifty-six studies were included. Most were retrospective cohorts using imaging or electronic health record data. Radiomics and deep learning dominated oncologic prediction, while structured clinical and laboratory data informed models for anesthetic risk, transfusion, and postoperative complications. Natural language processing (NLP) predicted ASA-PS classification from preoperative text. Only a small number of prospective or randomized studies were identified. AI-enabled CDSS shows promise for perioperative risk prediction and PAT triage, but most applications remain at the proof-of-concept stage. When prospectively validated and embedded in perioperative workflows, these tools could streamline preoperative work-ups, reduce unnecessary testing and day-of-surgery cancellations, and support safer intra- and perioperative monitoring. Prospective, multicenter validation and real-world implementation studies are therefore needed before routine clinical use.

**Supplementary Information:**

The online version contains supplementary material available at 10.1007/s10877-025-01404-w.

## Introduction

Preadmission testing (PAT) has become an essential component of modern perioperative medicine, ensuring that patients are optimized for surgery while minimizing unnecessary delays, cancellations, and complications. Traditionally, PAT has relied on clinician assessment, history taking, basic laboratory evaluation, and validated risk indices such as the American Society of Anesthesiologists Physical Status (ASA-PS) classification. Although these approaches provide a foundation for perioperative risk assessment, they are limited by interobserver variability, restricted sensitivity and specificity, and a growing mismatch between the rising complexity of surgical patients and the tools available to predict outcomes reliably [1–5]. In the context of rising surgical volumes and pressure to enhance efficiency, there is a critical need for improved methods of risk stratification and triage in PAT workflows.

Intra- and perioperative care generates high-frequency physiologic and laboratory data, yet clinicians must synthesize these signals in real time amid substantial cognitive load and alarm burden. Subtle, multivariate trends often precede hypotension, hypoxemia, arrhythmias, bleeding, delirium, or other complications, but are easily missed at the bedside. AI-enabled CDSS can in principle augment monitoring by fusing waveforms and electronic-health-record context to detect impending instability earlier, reduce non-actionable alerts, and standardize timely escalation—complementing, rather than replacing, clinician judgment.

Artificial intelligence (AI) and machine learning (ML) have emerged as promising approaches to augment clinical decision support systems (CDSS) across medicine. By leveraging large volumes of structured and unstructured perioperative data, AI-enabled CDSS has the potential to identify patterns and predictors of adverse outcomes that may be imperceptible to clinicians. In anesthesiology and perioperative medicine, these tools have been applied to a wide spectrum of tasks, including prediction of ASA-PS classification from electronic health record (EHR) narratives, forecasting of transfusion requirements, postoperative mortality risk prediction, and triage of patients for in-person versus remote PAT visits [6–9]. Imaging-based radiomics and deep learning models have been particularly prominent in oncology, where they are used for preoperative prediction of lymph node metastasis, tumor grade, and microvascular invasion (MVI) [10–12].

Recent reviews and pilot studies have further emphasized that AI systems can support anesthesiologists in preoperative evaluations, streamline resource allocation, and potentially reduce the burden of unnecessary tests [13, 14]. At the same time, systematic reviews of AI in neurosurgery and gynecologic oncology highlight a growing, but fragmented, literature, with heterogeneous methodologies and limited prospective validation [15, 16]. The application of wearable sensors and natural language processing (NLP) further expands the landscape of digital determinants of perioperative care, providing continuous physiologic monitoring and automated interpretation of free-text narratives [17].

Despite this rapid expansion, several limitations persist. Most AI-enabled CDSS remain at the proof-of-concept stage, with retrospective, single-center data dominating the literature. External validation, interpretability, and integration into PAT clinical workflows are rare. Importantly, the practical value of AI-enabled CDSS will differ across health systems: settings without stable electronic health records, reliable connectivity, or analytic support—in particular many low- and middle-income or financially constrained hospitals—may struggle to implement complex algorithms within their clinical information systems, risking a widening digital divide in perioperative care. This scoping review therefore aims to synthesize evidence from 2020 to 2025 on AI-enabled CDSS for preoperative assessment and risk stratification within PAT, map dominant approaches, highlight gaps in implementation, and identify opportunities for translation into routine perioperative care. We therefore focus on AI-enabled CDSS that inform preadmission decision-making and perioperative workflows in PAT. We include AI-CDSS evaluated within preadmission testing or preoperative evaluation clinics (PAT-embedded) and tools developed outside PAT but whose outputs directly inform preoperative risk stratification, triage, test stewardship, or planning (PAT-informative). The review protocol was preregistered on the Open Science Framework under a broader ‘digital determinants of health’ umbrella, and the scope was refined—prior to full data extraction—to concentrate on AI-enabled CDSS for feasibility and depth (see Methods for details).

## Methods

This scoping review followed the PRISMA-ScR (Preferred Reporting Items for Systematic Reviews and Meta-Analyses extension for Scoping Reviews) guidelines to ensure methodological rigor and transparency. The protocol was registered a priori with the Open Science Framework (OSF; DOI: 10.17605/OSF.IO/JKCRH). The original registration described a broad review of “digital determinants of health” in PAT. During piloting, we observed thematic saturation within AI-enabled CDSS and, for feasibility, refined the scope on August 23, 2025—before full data extraction—to focus on AI–enabled CDSS in PAT, while keeping databases, timeframe, and screening procedures unchanged. This refinement preserved the original aim by focusing on AI-driven tools that directly inform preadmission decision-making. The amendment, with justification and timing, is documented in the OSF record.

### Eligibility criteria

We included studies published between January 1, 2020, and August 1, 2025, that examined the use of AI, ML, or radiomics applied to preoperative evaluation, CDSS, or risk stratification in the context of PAT or closely related perioperative workflows. We restricted inclusion to publications from January 1, 2020 through August 1, 2025 to capture contemporary AI-CDSS development in the era of widespread EHR adoption and post–COVID-19 digital health acceleration, while keeping the scoping review manageable. Eligible study designs included randomized controlled trials, cohort studies (prospective and retrospective), cross-sectional analyses, diagnostic accuracy studies, modeling and radiomics studies, pilot or feasibility studies, quality improvement evaluations, usability or framework development studies, mathematical modeling, and systematic or narrative reviews. We focused on adult surgical populations and excluded pediatric-only cohorts and non-human or lab-only studies, as summarized in the title/abstract exclusion table (Supplementary Table S2). Articles were excluded if they (1) did not involve AI or CDSS, (2) addressed perioperative care outside the PAT setting without relevance to preoperative assessment, or (3) were published in a language other than English.

### Operational definitions and categorization rules

This review uses the following operational definitions for consistency. AI-enabled CDSS refers to any AI or ML approach intended to inform preoperative decisions—such as triage, risk prediction, test stewardship, or planning—within, or directly upstream of, PAT. Studies evaluated within PAT workflows are described as PAT-embedded, while tools developed outside PAT but directly usable for preoperative decision-making are described as PAT-informative. Primary reports are original data studies, including full manuscripts and conference abstracts; reviews include narrative, systematic, and meta-analytic syntheses. AI method families are recorded as regularized regression, tree-based, boosting, neural networks/deep learning, radiomics pipelines, NLP/large language models (LLMs), or hybrid approaches when methods are explicitly combined. Purely conventional, non-AI static risk scores without any AI/ML component were excluded. Validation tiers are recorded as internal split or cross-validation, external temporal or external site validation, prospective evaluation, or randomized designs; we report the highest tier achieved by each study. Dataset provenance is recorded as public or institutional, and multicenter indicates contributions from more than one clinical site. Hybrids are labeled in Table 1 with their components listed in-cell; each study is counted once in method-family and decision-function tallies, performance summaries cite the best validated model, conference abstracts are excluded from aggregated performance ranges, and reviews are counted separately from primary reports.

### Information sources and search strategy

A comprehensive search was conducted in PubMed, Embase, Scopus, and CINAHL for studies published between January 1, 2020, and August 1, 2025. Search strategies combined controlled vocabulary (e.g., MeSH and Emtree terms) with free-text keywords for “artificial intelligence,” “machine learning,” “clinical decision support,” and “preadmission testing” or “preoperative assessment.” Searches were limited to English-language publications. Database-specific strategies were adapted to indexing syntax, and the full search strings are provided in Supplementary file Appendix 1. Reference lists of included articles and relevant reviews were hand-searched for additional eligible studies.

### Selection process

All identified references were imported into Rayyan (Qatar Computing Research Institute) for deduplication, screening, and eligibility assessment [18]. Duplicates were removed automatically and confirmed by manual review. Two reviewers independently screened titles/abstracts against inclusion criteria, followed by full-text assessment with consensus resolution. Where a conference abstract and a subsequent full article described the same work, the full article superseded the abstract to avoid double counting. Counts for records, full-text reports, and included reports appear in the PRISMA flow and are summarized in Results.

### Data charting and variables

We used a predefined extraction schema (Supplementary Table S0) specifying variables, definitions, and coding rules, including study design; clinical domain; country and setting; sample size; dataset provenance (public versus institutional); multicenter status; data sources (EHR, laboratories, free text, imaging, physiologic monitoring, wearables); AI method family; primary decision-support function (risk prediction; triage/test stewardship; workflow optimization); validation approach (internal, external, prospective); reported performance (discrimination, calibration, decision impact); interpretability or explainability methods (e.g., feature importance, SHAP, saliency maps, or similar approaches when reported); deployment status; usability or patient/public involvement reporting; and integration characteristics with the EHR or monitoring systems.

### Handling studies with limited or difficult methodological appraisal

Consistent with PRISMA-ScR guidance for scoping reviews, we did not perform de-novo risk-of-bias assessments for primary studies and instead emphasized transparent description of study credibility. Two reviewers independently charted prespecified indicators (study design, dataset provenance and look-back, multicenter status, sample size, outcome definition, validation level [internal vs. external], calibration reporting [e.g., slope/intercept or O: E], operating thresholds, handling of missing data if stated, and evidence of deployment/EHR-embedded use). When a critical element was not reported or was ambiguous, we recorded it as “not reported (NR)” and did not infer a favorable status. In particular, if external validation could not be verified from the text, figures, or supplement, the study was coded as internal-only; if calibration or decision thresholds were not reported, they were coded NR and excluded from any maturity tallies that required those items.

Conference abstracts were eligible to map emerging domains and were treated as grey literature. They were charted with minimal fields, synthesized qualitatively, and used to illustrate trends, but they were not weighted in performance summaries or maturity counts; whenever a full article overlapped with an abstract, the full report superseded the abstract to avoid double counting. For systematic reviews, we applied AMSTAR-2 and reported overall confidence descriptively without excluding any studies; narrative and mini-reviews were marked “Not applicable (narrative)” (Supplementary Table S3). When multiple reports appeared to draw from the same cohort, we prioritized the most complete report for charting and noted related publications in the extraction codebook to avoid double counting. Disagreements were resolved by discussion and, when needed, senior review, using edge-case and priority rules prespecified in the data-extraction codebook (Table S0/S5).

### Synthesis of results

We used a narrative synthesis structured by primary decision-support function and by AI approach. Our approach follows PRISMA-ScR recommendations and is conceptually aligned with Arksey & O’Malley and JBI guidance for scoping reviews, emphasizing mapping and structured thematic synthesis rather than pooled effect estimation. A deductive codebook aligned to preadmission decision points (risk prediction; triage/test stewardship; workflow optimization; planning/imaging-driven support) was piloted and then applied by two reviewers with consensus resolution (Table S5). Each study was assigned one primary decision-support function for counting and domain synthesis, chosen by the authors’ stated objective and the main evaluated endpoint; when a study spanned multiple functions, secondary functions were recorded in the extraction schema but not double-counted. Edge-case priorities were prespecified: models evaluated on outcome probability were classified as risk prediction even if later used to route patients; workflow optimization required an operational endpoint (for example, time saved, automation accuracy, documentation effort) rather than only discriminative metrics; imaging or radiomics studies aimed at staging, invasion, or feasibility were classified as planning/imaging-driven support unless the primary endpoint was an outcome probability. We summarized validation tier (internal versus external; prospective/randomized), calibration, explainability, EHR/automation, and operational endpoints when reported. Hybrids are labeled in Table 1 with components listed in-cell; performance summaries cite the best validated model; conference abstracts were synthesized qualitatively only and were not included in aggregated performance ranges. Quantitative performance measures (for example, AUROC) were extracted where reported, and meta-analysis was not performed due to heterogeneity of designs, populations, and outcomes. For reports involving LLMs, we noted CHART-style transparency items relevant to reproducibility—model and version identifiers, prompt transparency and templates, input-context settings and parameters, evaluation datasets and protocols, availability of code or prompt materials, and any measures that facilitate replication (for example, seeds or fixed contexts)—and summarize these findings in the Discussion. No patients or members of the public were involved in the design, conduct, reporting, or dissemination plans of this scoping review. The topic was defined a priori by the investigator team, and the work synthesizes published literature only.

### PRISMA flow diagram

**Fig. 1:**
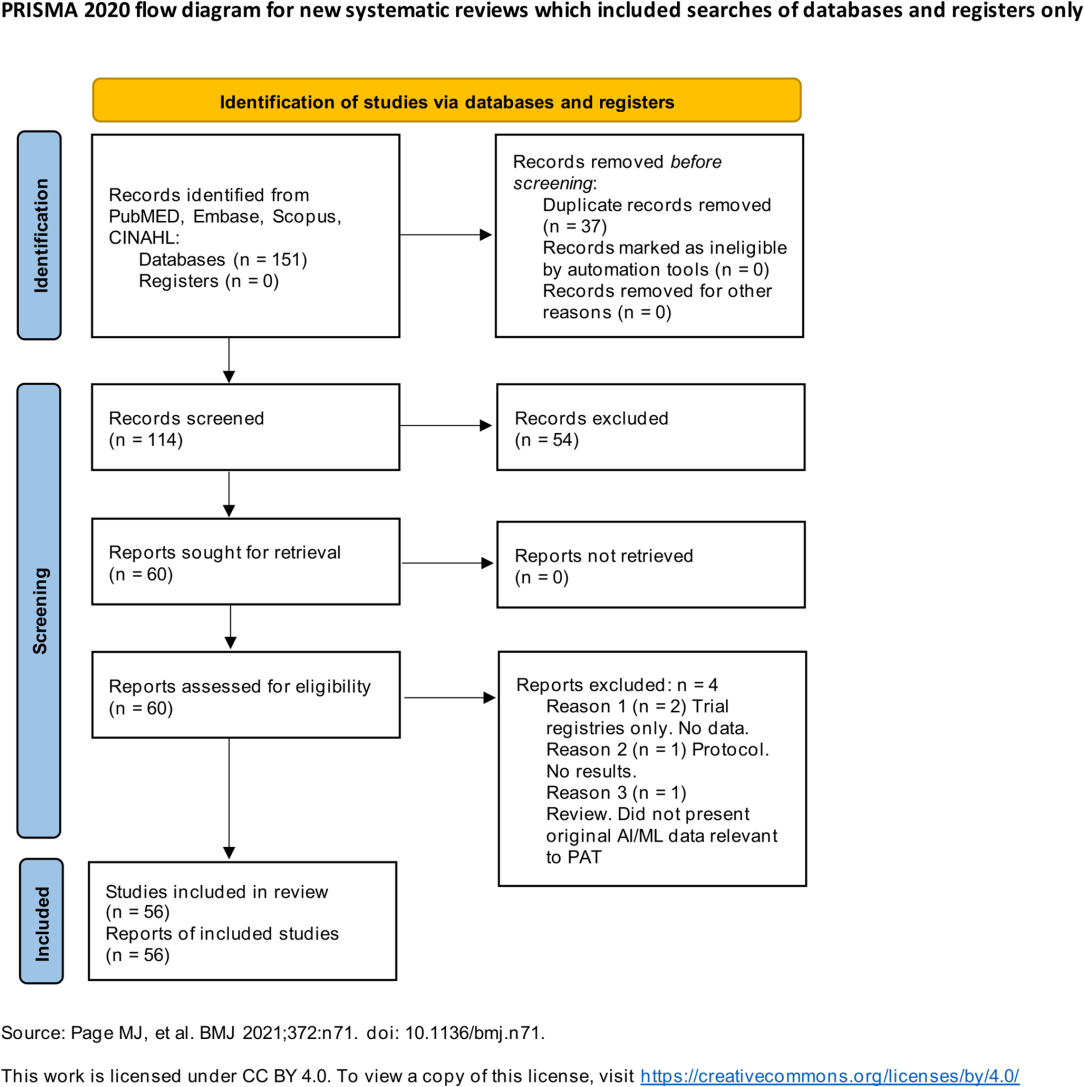
PRISMA 2020 flow diagram showing 114 records screened, 54 excluded at title/abstract, 60 full-text reports assessed, and 56 included (46 full manuscripts, 5 abstracts, 5 reviews)

The study selection process is summarized in a PRISMA 2020 flow diagram (Fig. 1). This diagram documents the number of records identified, screened, excluded, and ultimately included in the review [19].

## Results

### Search results and study characteristics

The database search yielded 151 records. After removal of 37 duplicates, 114 unique records were screened. Following title/abstract screening, 54 records were excluded. Sixty full-text reports were assessed; four were excluded (two trial registrations, one protocol, one review lacking AI/PAT focus). Fifty-six reports were included: 51 primary studies (46 full manuscripts and 5 conference abstracts) and 5 systematic/narrative/context reviews. Conference abstracts were mapped qualitatively only and were not included in any aggregated performance ranges. The PRISMA 2020 flow diagram (Fig. 1) summarizes the selection process.

### General study characteristics

The included studies spanned the years 2020 through 2025, reflecting the rapid expansion of AI applications in perioperative medicine during this time. Most included items were original research studies using retrospective datasets, pilot trials, or model development and validation designs. Five systematic or narrative reviews were also identified, adding perspective on current trends and challenges. In addition, five conference or supplement abstracts were included; these provide insight into emerging applications but lack the methodological transparency of full peer-reviewed articles. Study settings were diverse, including tertiary academic hospitals, multicenter collaborations, and national registry analyses. Geographically, the majority of studies originated from East Asia (China, Taiwan, Japan, South Korea), Europe, and the United States, underscoring a global interest in integrating AI into preoperative assessment workflows. A full list of included studies with details on study design, population, AI method, and outcomes is provided in Table 1. Terminology and categorization rules for AI-enabled CDSS, PAT-embedded versus PAT-informative studies, method families, validation tiers, handling of abstracts, hybrid models, dataset provenance, and multicenter status are defined in Methods and are applied consistently across Table 1 and the synthesis. Most identified tools were PAT-informative—developed outside dedicated preadmission clinics but directly usable to inform preoperative decision-making—with a smaller subset evaluated within explicit preoperative or perioperative medicine workflows (e.g., functional recovery prediction at preoperative assessments [17], delirium risk screening before cardiac surgery [20], a perioperative LLM CDSS trial [21], preoperative fitness prediction using wearables [22], and automated CHA₂DS₂-VASc scoring in preoperative evaluation [23]).

**Table 1:** Characteristics of included studies: Author, study Design, Population, AI Method, and outcomes

Ref #	First Author/Year	Study Design/Type	Population/ Setting	AI Method	Outcome/ Application	Dataset Provenance (public vs. institutional)	Multicenter (Y/*N*)
[10]	Eresen, 2020	Pilot study	Colon cancer patients undergoing surgery	ML (SVM radiomics)	Preoperative assessment of lymph node metastasis	Institutional	No
[11]	Zhang X, 2020	Two-center retrospective study	Hepatocellular carcinoma patients (CT radiomics)	Radiomics + ML classifier	Preoperative prediction of MVI status and risk stratification	Institutional	Yes
[15]	Buchlak, 2020	Systematic Review	Neurosurgical decision support literature	AI/ML review (classification, predictive models)	Synthesis of ML applications in neurosurgery CDSS	n/a	n/a
[24]	Kagerbauer, 2020	Retrospective Cohort (abstract)	70,000 non ICU surgical patients	Extreme Gradient Boosting (XGBoost)	Prediction of postoperative death/in-hospital mortality	Institutional	No
[17]	Bloomfield, 2021	Prospective observational/functional recovery	Knee replacement	ML + wearable sensors	Prediction of functional recovery set to realistic expectations	Institutional	No
[35]	Lu Y, 2021	Retrospective cohort	ACL reconstruction	ML (classification models)	Prediction of risk for overnight hospital admission	Public	Yes
[53]	Ramkumar, 2021	Retrospective cohort	Osteochondral allograft transplant	ML applied to imaging + patient factors (hybrid data)	Prediction of clinically meaningful outcomes and QoL after cartilage surgery	Institutional	No
[23]	van Giersbergen, 2022	Quality improvement evaluation	Preoperative patients requiring risk scoring	Automated CDSS for CHA₂DS₂-VASc	Accuracy of AI-derived scores vs. manual calculation; reduced clinician time	Institutional	No
[12]	Jiang, 2022	Multicenter retrospective study	Early-stage breast cancer patients	Radiomics (shear-wave elastography + ML)	Prediction of axillary lymph node status	Institutional	Yes
[31]	Assaf, 2022	Retrospective diagnostic model	Bariatric surgery candidates	ML classifiers	Pre-bariatric surgery diagnosis of hiatal hernia	Institutional	No
[36]	Hassan, 2022	Retrospective cohort	Patients after abdominal wall reconstruction	Novel ML approach	Prediction of hernia recurrence, surgical complications, and 30-day readmission	Institutional	No
[48]	Zhang F, 2022	Retrospective imaging study	Patients undergoing tracheal intubation	Deep learning + handcrafted features (hybrid features)	Automatic Mallampati classification, prediction of difficult intubation	Public (web images)	n/a
[27]	Bachelot, 2022	Retrospective model comparison (abstract)	Patients undergoing testicular sperm extraction	Supervised ML, multiple preprocessing pipelines	Prediction of successful sperm extraction; biomarker evaluation	Institutional	No
[6]	Wongtangman, 2023	Retrospective cohort (registry based)	Adult preoperative patients	Gradient boosting/ML ensemble	ASA-like risk score to flag patients for comprehensive preoperative screening	Institutional	Yes
[9]	Chen H, 2023	Retrospective cohort	Obstetric patients (cesarean sections)	ML prediction model	Prediction of intraoperative red blood cell transfusion requirement	Institutional	No
[8]	Chung P, 2023	Retrospective study	> 38,000 surgical patients	NLP models on preoperative notes	Prediction of ASA-PS classification	Institutional	Yes
[49]	Wu Y, 2023	Multicenter retrospective	Early-stage cervical cancer patients	Multimodal MRI radiomics	Prediction of lymphovascular space invasion	Institutional	Yes
[57]	Zhang X, 2023	Retrospective radiomics	Basilar artery occlusion patients treated with EVT	DWI-based radiomics	Prediction of post-treatment functional outcome	Institutional	Yes
[37]	Liu L, 2023	Retrospective cohort	Patients with gastric GIST tumors	Automated ML	Prediction of difficulty of endoscopic resection	Institutional	Yes
[38]	Chen X, 2024	Retrospective cohort	Patients with cesarean scar ectopic pregnancy	Interpretable ML model	Prediction of intraoperative hemorrhage risk	Institutional	Yes
[55]	Spear, 2023 (abstract)	Design & usability study	PAD patients/clinicians	User-centered design	Development of monitoring CDSS platform	Institutional	No
[7]	Tsai, 2024	Cross-sectional study	Gynecologic and obstetric surgical patients	ML risk stratification	Early detection of anesthetic risk	Institutional	No
[14]	Syversen, 2024	Narrative review	Wearable sensors in preoperative assessment	Review of sensor-based AI	Future applications in perioperative monitoring	n/a (review)	n/a (review)
[16]	Vasileva, 2024	Mini-review	Endometrial cancer MRI assessment	Review of AI methods	Early radiomic/deep learning applications, reproducibility issues	n/a (review)	n/a (review)
[20]	Sadlonova, 2024	Prospective observational (FINDERI)	Patients undergoing cardiac surgery	EHR-based screening tool	Prediction of postoperative delirium	Institutional	No
[34]	Yajima, 2025	Systematic review & meta-analysis	Older surgical patients	Mini-Cog cognitive test	Predictive ability for postoperative delirium	n/a (review)	n/a (review)
[40]	Yu Q, 2024	Retrospective model development	Older adults with hypertension	SHAP-based ML model	Prediction of preoperative acute heart failure	Institutional	No
[41]	Ganjouei, 2024	Retrospective study	Patients undergoing colectomy	ML predictive models	“Textbook outcome” prediction	Public	Yes
[42]	Wu J, 2024	Retrospective ML	Airway assessment patients	Multi-view metric learning	Prediction of difficult airway	Institutional	No
[65]	Xu H, 2024	Retrospective imaging study	Non-small cell lung cancer patients	Self-supervised pretrained ML + hyperbolic prototype embedding (hybrid method)	Prediction of occult lymph node metastasis	Institutional	No
[29]	Sudarsanam, 2024	Prospective observational study (abstract)	Patients undergoing major vascular surgery	Automated AI segmentation tool	Preoperative assessment of sarcopenia (frailty)	Institutional	No
[25]	Shukla, 2024 (abstract)	Secondary analysis/conference abstract	Elective cardiac surgery patients	Logistic regression ML model	Prediction of new-onset atrial fibrillation; diabetes as key predictor	Institutional	No
[58]	Raj, 2024	Mathematical model	Rhinoplasty patients	Quantitative modeling	Nasal deformity assessment for preop planning	Public	n/a (not site based clinical dataset)
[13]	Romito, 2025	Narrative review	Perioperative medicine, anesthesia & critical care	Review of AI/CDSS	Role of AI in preoperative assessment, surgical risk stratification	n/a (review)	n/a (review)
[26]	Nishibe, 2025	Retrospective cohort	Patients undergoing endovascular aortic repair	Decision tree ML	Prediction of 30-day mortality	Institutional	No
[28]	Peng, 2025	Multicenter retrospective	HCC patients, MRI-based	Radiomics model	Prediction of microvascular invasion	Institutional	No
[32]	Maradit Kremers, 2025	Retrospective EHR-based cohort	Patients undergoing total joint arthroplasty	ML risk modeling	Prediction of periprosthetic joint infection (PJI)	Institutional	No
[33]	Dragosloveanu, 2025	Retrospective	Patients with total joint arthroplasty	Supervised ML	PJI prediction models, validation	Institutional	No
[21]	Ke, 2025	Randomized crossover trial	Surgical patients (perioperative medicine)	LLM-based CDSS	Clinical & economic impact of periop LLM-CDSS	Institutional	No
[39]	Ghosh, 2025	Retrospective cohort	Lumbar spinal fusion patients	ML classification	Adverse outcomes prediction; anemia as key driver	Institutional	No
[43]	Lombaers, 2025	Model development	Endometrial cancer patients	Bayesian network (ENDORISK), hybrid data integrating molecular + imaging	Preop risk stratification; integration of molecular + imaging	Institutional	Yes
[22]	Hussain, 2025	Framework development	Surgical patients using wearables	Interpretable ML	Prediction of preop cardiorespiratory fitness	Institutional	No
[64]	Liu X, 2025	Retrospective cohort	Renal clear cell carcinoma patients	CT-based radiomics nomogram	Prediction of ISUP/WHO tumor grading	Institutional	Yes
[66]	Ge, 2025	Retrospective cohort	Elderly hypertensive patients with hip fractures	ML comparative models	Preop DVT incidence prediction	Institutional	No
[63]	Woodward, 2025	Dual-center cohort	Hip and knee replacement patients	AI risk stratification	Support for hub/ambulatory center pathways	Institutional	Yes
[47]	Patro, 2025	Prospective pilot	Adult cochlear implant patients	ML predictive model on multifaceted preoperative measures (hybrid data)	Prediction of implant outcomes	Institutional	No
[44]	Long, 2025	MRI-based radiomics study	Rectal cancer patients	Multiparameter radiomic	Prediction of metachronous liver metastasis	Institutional	No
[54]	Chen J, 2025	Retrospective cohort	Alloplastic breast reconstruction patients	AI risk prediction tool	Prediction of complications and outcomes	Institutional	No
[45]	Al Mopti, 2025	Retrospective imaging	Upper tract urothelial carcinoma patients	Radiomics of perirenal fat + ML	Staging and grading prediction	Institutional	No
[46]	Wang H, 2025	Radiomics/ ML	Mild cervical spondylotic myelopathy patients	MRI-based ML/radiomics	Spinal cord function assessment	Institutional	No
[50]	Zhang M, 2025	Embedding model development	Ophthalmic anesthesia patients	Predictive embedding models	Optimization of anesthesia in ophthalmology	Public	n/a (public dataset; not site derived)
[59]	Li R, 2025	Tool development	Cosmetic surgery patients	AI selection tool	Patient selection and risk-benefit balance	Institutional	No
[60]	Troian, 2025	Clinical evaluation	Patient education in lung cancer surgery	LLM (ChatGPT)	Accuracy of AI responses to patient questions	n/a (no patient dataset analyzed)	n/a for dataset
[61]	Luo, 2025	Retrospective study	Multi-disease CT reports	LLM benchmarking	Automated structuring of chest CT reports	Institutional	No
[30]	Rasenberg, 2025	Pilot crossover decision-support study	Pancreatic cancer surgery	CAD + 3D modeling	Decision support for resectability & vessel contact	Institutional	No (expert panel from 8 hospitals; patient cases from 1 center)

AI = artificial intelligence; ML = machine learning; SVM = support vector machine; NLP = natural language processing; CDSS = clinical decision support system; LLM = large language model; EHR = electronic health record; MRI = magnetic resonance imaging; CT = computed tomography; DWI = diffusion-weighted imaging; QoL = quality of life; CAD = computer aided detection; CHA_2_DS_2_-VASc = a stroke risk score for atrial fibrillation patients. Hybrids are labeled “hybrid” with component methods listed in the cell. Where a full article superseded an abstract, only the full article is counted. Conference and supplement abstracts were synthesized qualitatively only and are not included in performance ranges

Clinical contexts varied widely. Many studies employed radiomics and deep learning methods on CT, MRI, or PET/CT imaging to predict preoperative features such as lymph node status, MVI, or tumor grade. Others developed ML risk models for anesthetic or surgical outcomes, including prediction of postoperative complications, transfusion requirements, or mortality. A growing subset leveraged NLP and EHR data to automate perioperative risk stratification, including ASA-PS classification. Emerging areas included the use of AI-enabled CDSS, automated segmentation tools for sarcopenia or imaging markers, and pilot clinical trials testing AI-based workflows against usual care.

**Fig. 2:**
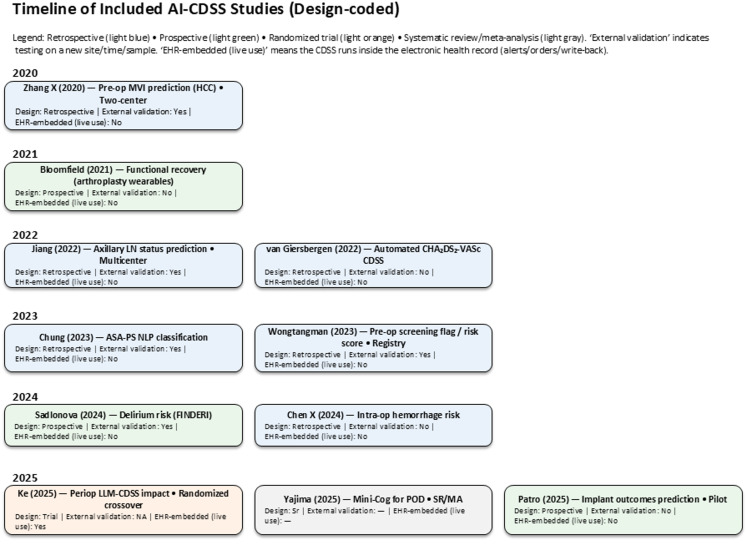
Timeline of artificial-intelligence–enabled clinical decision support systems (AI-enabled CDSS) in preadmission testing (PAT) and perioperative care. Years are shown from 2020 to 2025 along the left; boxes list each study’s design, external-validation status, and EHR-embedded (live-use) status. Study-design coding: Retrospective = analyses of existing data; Prospective (observational) = forward-looking data collection without randomization; Randomized trial (RCT) = randomized evaluation; Systematic review/meta-analysis (SR/MA) = secondary evidence synthesis. External validation means model testing on a new site/time/sample distinct from the training cohort. EHR-embedded (live use) means the CDSS runs inside the electronic health record (e.g., launches from the chart, surfaces alerts, writes back notes/orders); it is distinct from merely training on EHR data. Acronyms used in boxes: MVI = microvascular invasion; LN = lymph node; CHA₂DS₂-VASc = standard atrial-fibrillation stroke-risk score; ASA-PS = American Society of Anesthesiologists Physical Status; NLP = natural language processing; LLM = large language model; POD = postoperative delirium; SR/MA = systematic review/meta-analysis. Colors correspond to study design as indicated in the legend; Items are positioned by publication year; full citations and extraction details appear in Table 1 and the Supplement

Overall, the included body of literature demonstrates the breadth of AI approaches applied to PAT and perioperative evaluation. As shown in Fig. 2, these applications have progressed in a stepwise fashion, from early proof-of-concept models to recent randomized trials and multicenter validations.

### AI for preoperative risk stratification

Several studies explored anesthetic risk classification. Tsai et al. developed a LightGBM model in gynecologic/obstetric patients for ASA-PS classification (AUROC ≈ 0.68), illustrating feasibility rather than superiority to ASA-PS [7]. Chung et al. subsequently validated NLP models using free-text preoperative notes to predict ASA-PS class assignments across more than 38,000 patients, with performance comparable to manual clinician scoring and the added benefit of automated scalability [8]. A second analysis by Chung and colleagues applied transfer learning with Bio + Clinical BERT models—a type of transformer-based language model trained on biomedical and clinical text—confirming that text-based algorithms could replicate anesthesiologist-assigned ASA-PS classifications with AUROCs approaching 0.85 [8].

Beyond ASA-PS classification, ML has been leveraged to predict adverse perioperative outcomes. Several teams have explored prediction of in-hospital events in large surgical cohorts. Two conference abstracts reported preliminary models: Kagerbauer et al. described a ML approach in non-ICU surgical patients with high AUROC values (> 0.90) for in-hospital mortality prediction [24], and Shukla and Naik outlined a logistic regression–based model for new-onset atrial fibrillation after elective cardiac surgery, highlighting diabetes status, age, and postoperative complications as key predictors [25]. Nishibe et al. applied decision tree analysis in endovascular aortic repair and reported accurate early prediction of 30-day mortality, providing an example of ML applied in vascular surgery [26].

AI tools have also been used to predict resource-intensive perioperative events such as transfusion. Chen et al. trained a model using obstetric cesarean section data to estimate intraoperative red blood cell transfusion volume, achieving high predictive accuracy (96.8%) in distinguishing transfusion versus non-transfusion cases [9]. Bachelot et al. reported use of a random forest model for testicular sperm extraction outcomes, identifying hormonal markers such as inhibin B and follicle-stimulating hormone as potential predictors [27].

### AI in imaging and radiomics for preoperative evaluation

Imaging-based ML has become a prominent strand of preoperative evaluation, with radiomics and related approaches used to infer pathobiology, staging surrogates, and perioperative risk from routinely acquired CT, MRI, ultrasound, and hybrid imaging. A pilot support vector machine (SVM) radiomics model for colorectal cancer demonstrated that texture features extracted from preoperative CT improved prediction of nodal metastasis beyond traditional size criteria, underscoring the value of image heterogeneity as a biologic surrogate [10]. Hepatobiliary surgery has seen a parallel expansion: CT radiomics signatures have been trained to predict MVI in hepatocellular carcinoma, and combined clinico–radiomic classifiers have shown added prognostic stratification for recurrence and survival in preoperative cohorts [11]. More recently, multiphase MRI radiomics has been developed and clinically validated for MVI prediction with external testing, underscoring a trend toward multi-sequence modeling and generalizability beyond a single center [28]. Across these imaging studies, internal or external validation AUCs were generally in the moderate-to-high range (roughly 0.80–0.88 for nodal metastasis or MVI classification), although calibration and long-term impact on decision-making were less consistently reported.

Ultrasound-based radiomics and elastography are beginning to influence surgical axillary decision-making in breast cancer. A multicenter MRI/ultrasound elastography study showed that a shear-wave elastography radiomics signature, combined with sonographic node status and molecular subtype, achieved good discrimination for axillary lymph node burden and could separate low (1–2 nodes) from heavy (≥ 3 nodes) metastatic load in external validation—supporting more granular preoperative nodal triage [12]. Beyond disease biology, imaging AI has also targeted anatomic and body composition markers that inform perioperative risk. A fully automated deep learning tool segmented L3 skeletal muscle on preoperative CT for patients undergoing abdominal aortic aneurysm repair, producing sarcopenia indices in seconds with excellent agreement to manual ground truth; such automation enables scalable, objective sarcopenia/frailty assessment for surgical planning and PAT risk documentation [29].

AI-enabled anatomic modeling may also improve resectability assessment and preoperative confidence. In a crossover study using an integrated workstation, pancreatic head cancer experts reported higher perceived accuracy in gauging vessel contact and anatomic variants when 3D reconstructions and computer-aided detection (CAD) outputs were available in addition to CT, suggesting practical decision support at the tumor–vessel interface where small errors can alter surgical strategy [30]. Finally, outside oncology, ML models have been applied to focused preoperative classification problems, such as detecting hiatal hernia in bariatric candidates, where the primary aim was to refine diagnostic certainty before surgery [31]. These radiomics studies covered diverse domains including oncology, breast, vascular, and bariatric surgery.

### AI-Enabled clinical decision support systems for preadmission testing

AI-enabled CDSS are increasingly explored for perioperative assessment with direct relevance to PAT, but results vary across methods and settings. In a large EHR-based arthroplasty cohort, penalized regression (relaxed lasso) achieved the best discrimination and calibration for periprosthetic joint infection (PJI), with minimal or no incremental gain from more complex ML models [32]. In contrast, a single-center evaluation reported very high apparent performance for tree-based ML in PJI prediction, underscoring feasibility but also illustrating how model performance can depend on setting and analytic choices [33]. Taken together, current PJI studies suggest that model choice should be driven by calibration, external validation, and decision utility, rather than algorithmic complexity alone—an approach that aligns with PAT’s need for reliable thresholds and workflow fit. Reported AUROCs for these perioperative CDSS models typically ranged from the high 0.70s to low 0.90s in development and validation cohorts.

Cognitive complications such as delirium are also a growing concern in surgical patients. Sadlonova and colleagues validated a digital risk marker derived from EHR data for predicting postoperative delirium in cardiac surgery, demonstrating that EHR-based screening can be implemented before surgery using routinely collected clinical information [20].

Finally, the rapid emergence of LLMs introduces new opportunities for CDSS integration. Ke et al. reported that incorporating an LLM-based CDSS into perioperative medicine workflows improved both clinical and economic outcomes in a randomized crossover trial [21]. This example illustrates how AI-enabled tools can be embedded in preoperative and perioperative clinics to support documentation, risk stratification, and decision-making.

### Additional evidence and emerging directions

Several complementary studies provided broader context for the role of AI-enabled CDSS in preoperative care. Systematic and narrative reviews highlighted both the breadth and fragmentation of the literature. Buchlak and colleagues synthesized applications of ML in neurosurgical decision support, underscoring the methodological heterogeneity and lack of prospective validation across surgical specialties [15]. Vasileva et al. reviewed AI approaches to preoperative MRI in endometrial cancer, describing early radiomic and deep learning methods while emphasizing challenges in reproducibility [16]. Romito and Alexander further situated AI within anesthesiology and critical care, noting its potential to transform preoperative risk stratification while cautioning that implementation remains limited [13]. Syversen and colleagues added a digital health perspective, reviewing wearable sensors as preoperative assessment tools that may provide continuous physiologic monitoring in future perioperative pathways [14].

Exploratory applications were also reported. Assaf et al. applied ML to improve pre-bariatric surgery diagnosis of hiatal hernia, demonstrating that structured algorithms could enhance diagnostic certainty during preoperative evaluation [31]. Yajima and colleagues evaluated the Mini-Cog tool for predicting postoperative delirium in older adults with modest predictive ability [34].

### Outcome prediction and triage across surgical populations

Across diverse surgical domains, recent AI studies have moved beyond single-outcome surrogates to predict actionable events that matter for preadmission triage—disposition, composite “textbook” outcomes, procedure difficulty, and catastrophic intraoperative risks. In ambulatory orthopedics, ML identified patients at risk of unplanned overnight admission after anterior cruciate ligament reconstruction, based on perioperative factors available before surgery [35]. In general surgery, a multicenter team applied ML to predict hernia recurrence, complications, and 30-day readmission, reporting accurate performance across multiple outcomes [36].

Several studies emphasize predicting procedural difficulty as a key triage lever with direct operational consequences. Automated learning predicted difficulty for endoscopic resection of gastric gastrointestinal stromal tumors, supporting decisions about referral, equipment, and team readiness [37]. In obstetrics, an interpretable ML model predicted the risk of intraoperative hemorrhage during surgery for cesarean scar ectopic pregnancy, with performance explained using explicit feature contributions [38]. For spine surgery, an ML analysis identified preoperative anemia as a strong driver of adverse outcomes following lumbar procedures [39].

Models also targeted broader medical risk. In older adults with hypertension, a preoperative acute heart failure model incorporated SHAP (Shapley Additive Explanations) and permutation feature importance to provide transparent attribution of predictive features [40]. Ganjouei et al. reported on the development of a ML model for “textbook outcome” in colectomy, defined as a composite measure of uneventful recovery [41].

Anesthesia-specific triage targets were likewise advanced. Wu et al. developed a multi-view metric-learning model using preoperative facial photographs to predict difficult airway (Cormack–Lehane III–IV), achieving ~ 78% accuracy with sensitivity ~ 83% and specificity ~ 76% on 5-fold cross-validation [42]. Beyond single domains, Bayesian network approaches (e.g., ENDORISK) integrate molecular and clinicopathologic data for preoperative risk stratification in endometrial cancer [43]. Hussain et al. developed an interpretable framework using wearable data to predict preoperative cardiorespiratory fitness [22].

## Discussion

### Trends

Radiomics and imaging-based studies continue to dominate the field [10–12, 28–31, 44–46], but there is growing attention to EHR-driven decision support and early pilot implementations of AI-enabled CDSS [6, 8, 20, 21, 23, 32, 33, 47]. A substantial proportion of included investigations applied ML, deep learning, or NLP to enhance preoperative risk stratification, in some cases outperforming traditional clinical scoring systems [7, 32]. Collectively, these studies illustrate how imaging-centric AI can enrich preoperative evaluation: (1) as biological surrogates that inform risk and prognosis (e.g., nodal disease, MVI); (2) as anatomic and body composition quantifiers that scale frailty and technical risk assessment; and (3) as decision aids for complex surgical planning. Most reports remain retrospective and single-center, with ongoing challenges in segmentation, feature reproducibility, and external validation limiting generalizability. Nevertheless, the overall signal is consistent—AI can provide clinically relevant information beyond standard reads, and emerging work increasingly incorporates external cohorts, multimodal inputs, and workflow-adjacent decision support. Because discrimination and calibration metrics were reported most consistently for preoperative risk-stratification models, we summarized AUROC ranges quantitatively in that subsection; in other domains, performance metrics were too heterogeneous for meaningful aggregate ranges and are therefore synthesized qualitatively.

### Gaps

Despite this momentum, important gaps in the evidence base remain. Most models are still developed using retrospective cohorts, often with exploratory or convenience sampling, and relatively few have undergone prospective or multicenter evaluation. External validation is inconsistently reported, calibration and decision thresholds are frequently absent, and real-world impact on perioperative workflows, cancellations, or patient outcomes is rarely measured. Many reports focus on narrow surgical domains or single institutions, with limited attention to equity, variation in PAT structures, or downstream consequences for resource allocation. In addition, most models were derived from tertiary centers or closely linked health systems in East Asia, Western Europe, and North America, with relatively few public datasets and minimal representation of low-resource settings with limited information technology infrastructure. This concentration of institutional and geographic provenance limits generalizability and may underestimate context-specific challenges for perioperative assessment in under-resourced environments.

### Implications for practice

**Fig. 3:**
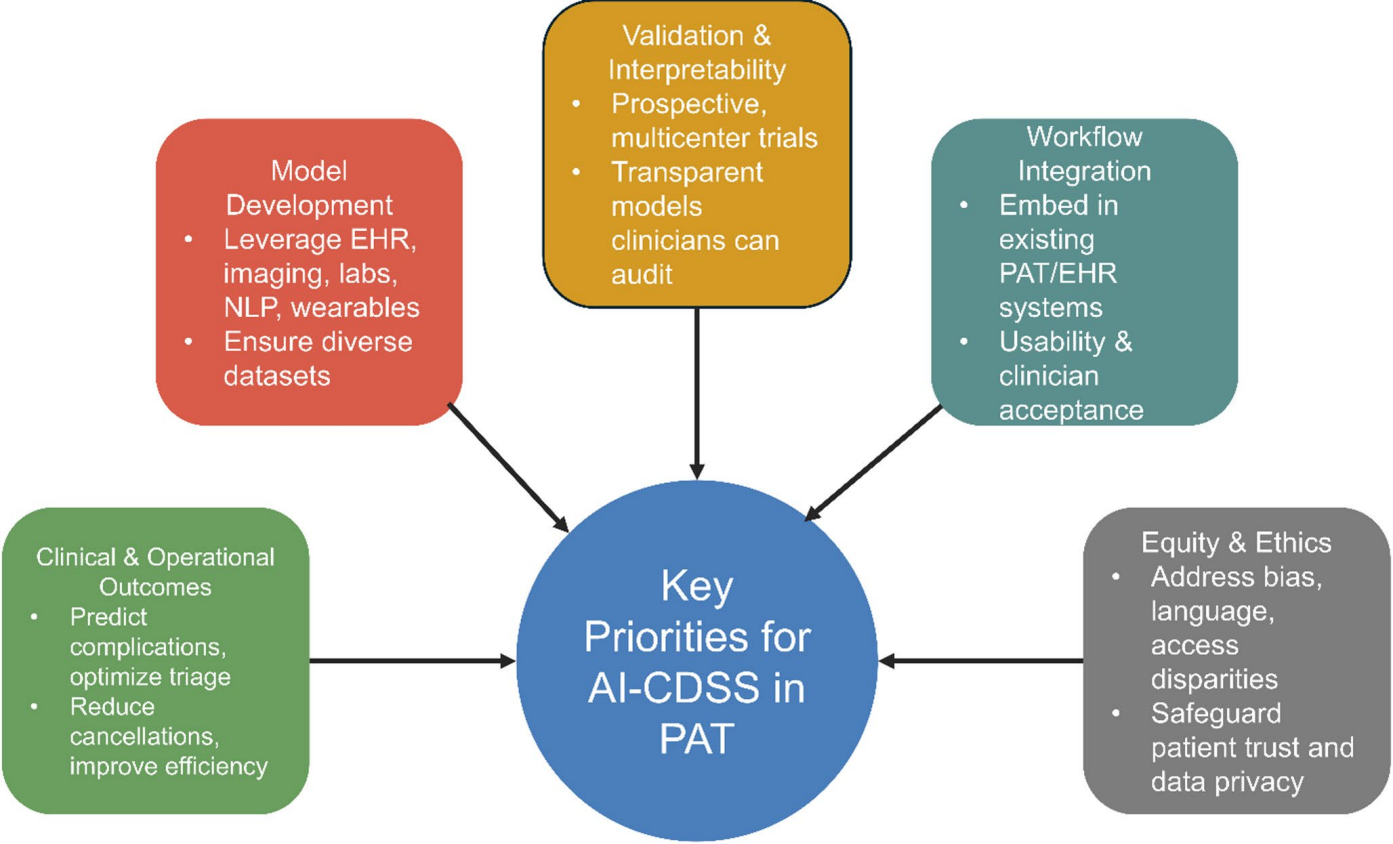
Key priorities for implementing artificial intelligence–enabled clinical decision support systems (AI-CDSS) in preadmission testing (PAT). The framework highlights five domains essential for translation into practice: (1) model development using diverse perioperative data sources, (2) validation and interpretability through prospective, multicenter trials and transparent methods, (3) workflow integration with existing PAT and electronic health record systems, (4) equity and ethics to address bias, language, and access disparities, and (5) clinical and operational outcomes such as complication prediction, triage optimization, and efficiency gains

AI model development for preoperative risk stratification has grown rapidly, yet translation into routine practice remains limited. By integrating structured variables such as laboratory values and comorbidities with unstructured information from clinical notes and imaging, these approaches generate more nuanced and scalable risk prediction tools than traditional indices [6–8, 10–12, 28, 46, 48]. Although most analyses remain retrospective, consistent gains in predictive accuracy indicate that AI-enhanced PAT could enable earlier identification of high-risk patients and more efficient triage once validated [7–12, 28, 32, 49]. Outcome prediction is now being explored across multiple surgical domains, including orthopedics, general surgery, gastrointestinal surgery, obstetrics and gynecology, spine surgery, anesthesiology, and gynecologic oncology [22, 35–37, 39, 43, 50]. Shared features—such as composite endpoints, emphasis on interpretability, and explicit linkage to modifiable risks—make these models particularly suited to PAT, where the clinical objective extends beyond prediction to actionable rerouting of patients by venue, monitoring intensity, optimization strategies, and surgical timing. Despite this promise, several barriers still constrain integration into routine perioperative workflows. Figure 3 summarizes five domains that will be critical for successful implementation of AI-enabled clinical decision support systems in PAT. Recent work by Eyth and colleagues on the TRANSFUSE risk model exemplifies this principle: a regression-based instrument, derived from large surgical cohorts, achieved excellent discrimination for intraoperative blood transfusion and performed comparably to multiple ML–derived scores, while remaining usable without specialized AI infrastructure [51]. Future implementation studies—including within residency and fellowship programs—should quantify not only clinical impact but also how AI-enabled CDSS affect documentation burden, triage time, and training: for example, estimating ‘numbers needed to benefit’ in terms of prevented perioperative complications or avoided day-of-surgery delays, and mapping how acceptance evolves as clinicians gain experience with these tools [52]. Such designs provide a template for PAT-focused CDSS that can bridge high- and low-resource environments. For intra- and perioperative monitoring, AI-enabled CDSS should be framed as signal-fusion tools that flag impending instability and support reproducible responses, rather than automated treatment engines. Because external validation and EHR-embedded deployments were rare, clinical adoption hinges on prospective validation, calibration/threshold transparency, and integration with alarm governance, audit logging, and rollback plans. Standardization of input features, outcome definitions, and alert-threshold logic across vendors and institutions will also be essential to support safe, reproducible AI-PAT applications.

### Workflow integration and clinical usability

A critical question in translating AI from proof-of-concept to practice is whether models can be realistically embedded in perioperative workflows. Several studies illustrate both opportunities and challenges. Ramkumar and colleagues used ML to analyze preoperative imaging and patient factors in osteochondral allograft transplantation, demonstrating that integrating multimodal data could improve prediction of clinically meaningful outcomes [53]. While accurate, such models depend on specialized imaging and registries not consistently available at all centers.

Airway management is another area where usability is paramount. Zhang et al. developed an automated model for Mallampati classification to predict tracheal intubation difficulty, showing that simple preoperative photographs could be processed into reliable airway risk assessments [48]. This represents a workflow-aligned approach that leverages data already collected at PAT, minimizing disruption to standard practice.

In oncology, Long and colleagues built a multiparameter MRI radiomics model to predict metachronous liver metastasis in rectal cancer, with implications for triaging patients to intensified surveillance or neoadjuvant strategies [44]. Chen and colleagues extended AI into plastic surgery, applying risk prediction to alloplastic breast reconstruction, where perioperative complications carry both functional and cosmetic consequences [54]. Similarly, Al Mopti et al. demonstrated that radiomics of perirenal fat could support renal cell carcinoma staging, though its translation depends on reproducibility across scanners and radiology protocols [45]. Together, these examples highlight that usability is determined not only by predictive accuracy, but also by alignment with available data streams, ease of integration, and clinical relevance. AI systems that build upon routinely collected preoperative data — such as EHR fields, airway photographs, or standard imaging — are more likely to gain adoption in PAT settings than those requiring specialized acquisition or high-resource infrastructure. These usability considerations naturally set the stage for defining what the next phase of research should prioritize.

### Future directions: prospective Validation, emerging Applications, and multimodal expansion

The next phase of AI-enabled CDSS in PAT will depend on pragmatic validation, human-centered design, and expansion into underexplored clinical domains. Several unique contributions in the recent literature illustrate these trajectories. One direction is the pragmatic embedding of AI within clinical workflows. A quality-improvement evaluation of an automated CDSS found that AI-derived CHA₂DS₂-VASc scores were as accurate as manual calculation but required less clinician time, highlighting how targeted automation can reduce cognitive load in PAT [23]. Spear et al. described a user-centered design process for a monitoring platform in peripheral arterial disease, emphasizing alignment of AI outputs with clinician and patient needs [55].

Multicenter radiomics studies are also emerging as examples of prospective readiness. A collaborative analysis used multimodal MRI to assess lymphovascular space invasion in cervical cancer, demonstrating improved discrimination when radiomics features were combined with clinical data [49]. This kind of integration suggests a path forward for radiomics: rather than functioning as a stand-alone tool, radiomic signatures may achieve the most clinical value when embedded alongside standard clinical variables. Importantly, the multicenter design also indicates that these approaches can be validated across diverse institutions, moving them closer to real-world adoption. Similarly, MRI-based models have been trained to predict CD3 expression in cervical cancer, signaling a pathway for AI to integrate imaging with immunologic and pathologic markers in surgical oncology [56]. Additional radiomics work has targeted acute neurologic disease: diffusion-weighted MRI features predicted outcomes after basilar artery occlusion treated with endovascular therapy, broadening perioperative AI applications beyond oncology into neurology [57]. This extension underscores that perioperative AI is not confined to cancer staging or surgical planning but can also inform prognostication in acute, high-stakes conditions. By demonstrating feasibility in cerebrovascular disease, these studies highlight the potential for AI tools to bridge traditionally separate domains of perioperative and neurologic care, and they point toward future models that may assist in cross-disciplinary triage and recovery planning. Spinal applications have also been explored; radiomics and ML were used to assess spinal cord function in mild cervical spondylotic myelopathy, offering a novel surrogate for surgical planning and risk stratification [46]. This line of work illustrates how AI can generate entirely new functional biomarkers from standard imaging, providing insight into physiologic reserve that is not otherwise captured in routine preoperative evaluation. If validated prospectively, such biomarkers could complement conventional risk indices, enabling more individualized triage of spine patients and offering a template for similar approaches in other surgical domains.

Emerging specialty applications further illustrate the breadth of AI in perioperative medicine. Zhang and colleagues applied predictive embedding models to optimize ophthalmic anesthesia, demonstrating how AI approaches may be adapted for highly specific subspecialty contexts [50]. Beyond simply tailoring drug choice or anesthetic depth, this work signals how embedding methods can capture nuanced physiologic and procedural features unique to ophthalmic surgery. Such adaptability suggests that AI-enabled CDSS could be extended to other niche surgical areas where patient populations, procedural risks, and outcome measures differ substantially from general perioperative cohorts. If validated and generalized, these embedding-based strategies may provide a blueprint for developing specialty-specific CDSS that augment anesthesiologist expertise while maintaining alignment with unique workflow requirements.

Emerging domains extend AI into aesthetic and patient-centered surgery. A model quantifying nasal deformities was proposed to augment preoperative assessment in rhinoplasty, offering objective metrics to complement traditional visual inspection [58]. Other cosmetic applications include an AI tool to support patient selection in elective cosmetic surgery, designed to balance surgical risk with expected benefit [59]. These examples demonstrate that perioperative AI is not confined to high-risk surgical populations but can also contribute to specialties where outcomes hinge on aesthetics, patient satisfaction, and shared decision-making. By providing standardized, quantifiable metrics, AI has the potential to reduce subjectivity, support transparent patient counseling, and introduce new levels of rigor into areas of surgery where patient perception and expectations are central.

Another frontier is the incorporation of LLMs into perioperative contexts. A preliminary evaluation tested ChatGPT’s responses to common patient questions about lung cancer surgery. Findings were variable, illustrating both the potential and the limitations of LLMs in patient-facing perioperative communication [60]. Complementary work benchmarked LLMs in structuring chest CT reports across multiple disease domains, highlighting both the promise of automating synthesis and the risks of error propagation in critical care settings [61]. Taken together, these early efforts underscore the disruptive potential of LLMs: they could expand perioperative care beyond prediction to include real-time communication, documentation, and information synthesis. At the same time, variability in accuracy and the potential for unchecked errors emphasize that LLMs must be deployed cautiously, with human oversight and domain-specific fine-tuning. If addressed systematically, these challenges position LLMs as a future class of perioperative CDSS capable of engaging both clinicians and patients directly.

## Limitations

This scoping review maps recent literature on AI-enabled clinical decision support systems relevant to PAT, but several limitations should be acknowledged. Our restriction to English-language publications may under-represent AI-CDSS research from non-English-speaking countries, including substantial AI work from China, Korea, and other regions, and may bias the geographic distribution of included models. Our focus on AI- and ML-based decision support within PAT and closely related perioperative triage contexts means that broader digital health interventions and non-AI tools are not represented, so the review does not capture the full spectrum of digital determinants of preadmission testing.

Consistent with PRISMA-ScR guidance, we did not perform formal risk-of-bias assessments for primary studies and instead emphasized breadth and conceptual mapping rather than graded certainty. We did not systematically apply TRIPOD-AI, CONSORT-AI, or DECIDE-AI checklists to each study; instead, we charted credibility and reporting indicators (model description, validation tier, calibration, thresholds, deployment context, and, for LLMs, CHART-style transparency) that align with key domains from these frameworks. Systematic and narrative reviews were appraised descriptively using AMSTAR-2 without excluding any studies, and findings were synthesized narratively rather than meta-analytically because of substantial heterogeneity in patient populations, predictors, outcomes, and validation strategies. Our operational categorization rules—distinguishing PAT-embedded from PAT-informative tools, assigning a single primary decision-support function per study, and labeling hybrid models by their dominant method family—inevitably simplify more complex workflows. Although these rules were prespecified and applied consistently, misclassification or overemphasis of certain domains remains possible.

In addition, the reporting quality of the underlying literature imposes constraints on our synthesis. Several included items were conference abstracts, which we treated as grey literature and synthesized qualitatively only; they were not incorporated into aggregated performance ranges or maturity tallies, and any corresponding full articles superseded earlier abstracts to avoid double counting. Many primary reports incompletely described calibration, decision thresholds, deployment context, or workflow integration, limiting our ability to comment on real-world impact. For reports involving LLMs, transparency across key reporting domains was variable: Ke et al. [21] identified the model family and deployment platform and described a long-context prompting workflow but did not provide full prompt templates, seeds, or decoding parameters; Troian et al. [60] specified the model and evaluation task but did not share complete prompts or parameterization needed for exact replication; Luo et al. [61] compared several models and task formats and reported performance, yet prompt content, context settings, and code or prompt materials were only partially disclosed and reproducibility artifacts were not provided. These omissions limit independent replication and external auditing and motivate more standardized, CHART-style reporting of perioperative LLM tools. Taken together, these methodological and reporting limitations temper the strength of inferences that can be drawn from the current evidence base and reinforce the need for more complete, prospective, multicenter evaluations in this space.

### Implementation barriers

Beyond technical performance, deployment of AI-enabled CDSS in PAT should follow emerging ethical frameworks for health AI that emphasize transparency, accountability, and relational aspects of clinician–patient interaction, such as the Belmont-inspired ‘relational AI’ perspective articulated by Sim and Cassel [62]. Bringing AI-enabled CDSS from promising performance to dependable use in PAT depends on resolving several implementation barriers. At the technical layer, the most consistent risks are transportability and calibration: models trained on narrow samples or single devices degrade under new case-mix, scanners, or documentation styles, and performance reported as AUC rarely translates to thresholded decisions without local calibration to prevalence and operating points. Data lineage and missingness handling are often under-specified, which complicates safe automation. Interpretability was also variably reported; while some models provided feature importance or SHAP-based explanations, many functioned as essentially opaque “black boxes,” which can limit clinician trust and slow adoption in preadmission workflows. Integration into the EHR matters as much as model accuracy; systems that sit outside order sets, note templates, or scheduling workflows do not change behavior even when technically sound, and the lack of real-time interoperability or write-back can strand tools at the pilot stage. Post-deployment monitoring is rarely specified; without drift detection, error logging, and rollback plans, models can silently degrade as practice or patient mix changes. Across the 56 reports, transportability and calibration were the dominant technical gaps: external validation was described in only a subset of multicenter or externally tested studies [12, 43, 63], and thresholded calibration suitable for local decision points was rarely reported [32, 38, 43]. For example, Liu et al. developed a CT-based radiomics nomogram for renal clear cell carcinoma tumor grading but explicitly noted that generalizability across scanners and institutions remained a key hurdle [64]. Xu et al. similarly demonstrated improved prediction of occult nodal metastasis in non-small cell lung cancer using self-supervised and hyperbolic representations, yet the architecture and training requirements make routine deployment challenging in typical perioperative workflows [65]. Workflow embedding was also limited; where integration or automation signals were described, they were largely confined to automated scoring or documentation assistance rather than write-back into order sets or scheduling workflows [21, 23]. Operational endpoints were seldom measured beyond time saved or documentation effort, with few studies linking model use to triage accuracy or routing utility [21, 23, 63]. For LLMs specifically, prompt transparency, version identifiers, parameter and context settings, and reproducibility artifacts were variably missing across the included LLM papers, constraining auditable deployment in clinical pathways [21, 60, 61].

Organizational factors are at least as important. Preadmission workflows are tightly coupled to clinic schedules, testing slots, and downstream operating room constraints, so tools that predict risk but do not specify who acts, when, and how tend to stall. Clinicians need clear thresholds that map to actions such as phone screen versus in-person PAT or escalation to an anesthesia consult, along with documented accountability for exceptions. Training requirements, cognitive load, and user experience determine adoption; alert fatigue and duplicate data entry quickly erode trust. Information services capacity and competing priorities can delay integration for months, and financial cases are often unproven unless time saved, cancellations avoided, or unnecessary tests reduced are measured prospectively. Equity obligations add further needs for subgroup performance reporting and mitigation plans when calibration differs across age, sex, language, or comorbidity strata [6, 21, 23, 63]. Ge et al.’s model for preoperative deep vein thrombosis in elderly hypertensive hip fracture patients exemplifies this challenge, as the cohort had limited racial and socioeconomic diversity despite strong apparent performance [66].

Regulatory and governance expectations are tightening for perioperative AI. Even when not marketed as devices, CDSS benefit from device-like discipline: traceable datasets and versioning, change control, pre-specified operating thresholds, and documented post-deployment monitoring. Privacy and data-sharing constraints limit multi-site retraining or cross-institution model evaluation. For LLM-based systems, reproducibility depends on transparent model and version identifiers, prompt and context documentation, and guardrails to reduce hallucination and protected health information leakage; these artifacts were variably absent across the LLM reports in this review, which limits auditable use in a clinical pathway [21, 60, 61].

### Common failure points

Across the corpus, failures cluster around a few patterns: outcome-only reporting without action thresholds or local calibration; single-center imaging or note-based models that break under domain shift; prototypes evaluated outside the EHR with no clinician-in-the-loop plan; dashboards that measure AUC but not time saved, triage accuracy, or cancellations prevented; and LLM studies that name a model but omit prompt templates, parameters, and seeds. The studies that moved closest to implementation specified the target decision and operating threshold, measured an operational endpoint such as documentation time or automated scoring time, disclosed how the tool would live inside the EHR, and evaluated performance externally or prospectively. Future AI-enabled CDSS intended for PAT should therefore pair discriminative performance with calibrated, thresholded decisions, prospectively measured workflow impact, explicit EHR integration steps, and a monitoring plan that tracks drift and subgroup calibration over time [12, 21, 23, 32, 57, 60, 61].

## Conclusion

This scoping review demonstrates that AI-enabled CDSS in PAT and perioperative care have advanced rapidly over the past five years. Radiomics and imaging-based approaches remain dominant, but the field is diversifying with growing emphasis on EHR-driven models, NLP, and early pilot implementations of CDSS. Across domains ranging from orthopedics to oncology, AI systems have shown that they can match or surpass traditional clinical scoring methods in predictive performance. Collectively, these trends highlight a consistent signal: AI has the capacity to enrich perioperative evaluation by providing biologic, anatomic, and functional insights that extend well beyond conventional assessment.

A pragmatic roadmap for bringing these tools into routine PAT workflows requires at least three steps. First, candidate AI-enabled CDSS should undergo prospective, multicenter validation with transparent calibration, fairness, and subgroup analyses. Second, systems that demonstrate value must be integrated with EHRs and perioperative monitoring platforms, with attention to alarm governance, auditability, and clinician usability. Third, pragmatic trials and implementation studies are needed to quantify effects on perioperative efficiency and safety, including unnecessary testing, day-of-surgery cancellations, complications, and patient experience. Together, these steps can help translate promising AI-enabled CDSS prototypes into dependable tools that improve the everyday work of PAT.

At the same time, important shortcomings persist and shape future priorities. As discussed, much of the current evidence remains retrospective and single-center, with limited external validation and inconsistent attention to transparency, equity, and workflow integration. Moving forward, prospective multicenter studies, multimodal integration of radiomics, pathology, laboratory, and clinical data, and rigorous user-centered design will be essential. Usability and workflow alignment will determine whether AI-enabled CDSS can move from proof-of-concept to daily practice, with the greatest promise seen in models that leverage routinely collected data. Expanding applications into underexplored specialties—including neurology, spine, aesthetics, and patient-facing communication via LLMs—underscores that perioperative AI is not a narrow innovation but a broadly adaptable paradigm. Future research must emphasize inclusive sampling, standardized reporting, and pragmatic evaluation to ensure that AI-enabled CDSS in PAT improve outcomes for all surgical populations.

## Supplementary Information

Below is the link to the electronic supplementary material.

Supplementary material 1 (DOCX 34.0 kb)

## Data Availability

All data generated or analyzed during this study are included in this published article and its supplementary information files.
