# Supplementary material for: Artificial intelligence-enabled clinical decision support systems in preadmission testing: a scoping review of risk prediction, triage, and perioperative workflows (2020–2025)
Source: J Clin Monit Comput. 2026 Jan 31;40(2):525–45. doi: 10.1007/s10877-025-01404-w (PMC13053387; doi:10.1007/s10877-025-01404-w)
Supplement: Supplementary file 1 — Supplementary material 1 (DOCX 34.0 kb) [file 10877_2025_1404_MOESM1_ESM.docx]

Supplementary Appendix 1. Full Search Strategies

- PubMed (searched August 23,2025)

("artificial intelligence"[Mesh] OR "machine learning"[Mesh] OR "deep learning"[Mesh]

OR "natural language processing"[Mesh] OR "artificial intelligence"[tiab] OR … )

AND ("clinical decision support systems"[Mesh] OR "decision support"[tiab] … )

AND ("preadmission testing"[tiab] OR "preoperative evaluation"[tiab] OR … )

AND ("2020/01/01"[Date - Publication] : "2025/08/01"[Date - Publication])

- Embase (Ovid; searched August 23, 2025)

('artificial intelligence'/exp OR 'machine learning'/exp OR 'deep learning'/exp … )

AND ('clinical decision support system'/exp OR 'clinical decision support':ti,ab … )

AND ('pre-admission testing':ti,ab OR 'preoperative care'/exp OR … )

AND [2020–2025]/py

- Scopus (Elsevier; searched August 23,2025)

(TITLE-ABS-KEY("artificial intelligence" OR "machine learning" OR … ))

AND (TITLE-ABS-KEY("clinical decision support" OR "CDSS"))

AND (TITLE-ABS-KEY("preadmission testing" OR "preoperative care" OR … ))

AND PUBYEAR > 2019 AND PUBYEAR < 2026

- CINAHL (EBSCO; searched August 23, 2025)

(MH "Artificial Intelligence+" OR TI "machine learning" OR AB "deep learning" … )

AND (MH "Decision Support Systems, Clinical+" OR TI "CDSS" OR AB "decision support")

AND (MH "Preoperative Care+" OR TI "preadmission testing" OR AB "preoperative evaluation")

Limiters: Published Date 20200101–20250801; English Language

*Table 2 Database searches and results (2020–2025)*

| **Database** | **Platform** | **Coverage** | **Date searched** | **Results retrieved** |
| --- | --- | --- | --- | --- |
| PubMed | NCBI | 2020–2025 | Aug 23,2025 | 6 |
| Embase | Ovid/Elsevier | 2020–2025 | Aug 23,2025 | 48 |
| Scopus | Elsevier | 2020–2025 | Aug 23,2025 | 93 |
| CINAHL | EBSCO | 2020–2025 | Aug 23,2025 | 4 |

*Results reflect database contents as of August 23, 2025. Full electronic search strategies for all databases, including exact Boolean operators and limits, are provided in Supplementary Appendix 1*

**Supplementary Table S0. Data dictionary (variables and definitions)**

*Table S 0 Data dictionary (variables and definitions)*

| Variable | Definition | Coding options/rules |
| --- | --- | --- |
| Ref # | Reference number used in manuscript | Integer (1–60) |
| First author_(Year) | First author surname and publication year | Text (e.g., “Wongtangman 2023’) |
| Study_design_type | Design label (RCT, prospective cohort, retrospective, cross-sectional, diagnostic, modeling, QI, usability, framework, mathematical, review, abstract) | Controlled vocabulary |
| Setting & population | Clinical setting and population descriptors | Free text short phrase; e.g., 'adult surgical candidates, PAT clinic' |
| Country/region | Country or multi-country designation | ISO country or region name |
| Sample size (N) | Total participants/records analyzed | Integer |
| Dataset provenance | Source of data | Public / Institutional |
| Multicenter | More than one clinical site contributed data | Yes / No |
| Data sources | EHR, labs, free text, imaging, physiologic monitoring, wearables | Multi-select list |
| Ai method family | Primary analytic approach | LR/LASSO, RF, XGB, SVM, DL?CNN, LLM?NLP, BN, NB, KNN, Ensemble, Other |
| Primary CDSS function | Intended decision-support purpose | Risk prediction; Triage/test stewardship; Workflow optimization; Planning |
| Validation tier | Highest level of validation reported | Internal split/CV; External temporal; External site; Prospective; RCT |
| Outcome/application | Clinical endpoint or task | Free text (concise) |
| Performance (discrimination) | AUC/C-index and CI if available | Numeric with CI |
| Performance (calibration) | Calibration slope/intercept or H-L test | Reported / Not reported + details |
| Explainability | Use of SHAP, LIME, interpretable NB, feature importance | Reported / Not reported + method |
| EHR integration/automation | Evidence of EHR linkage or automated scoring | Yes / No + brief |
| Operational endpoint | Time savings, triage accuracy, DOS delays, LOS, etc | Reported / Not reported + metric |

*Data extraction schema (codebook) Variables, definitions, and coding rules used during charting—these terms are referenced in Table 1 and the Results (e.g., dataset provenance, multicenter status, AI method family). AI method family acronyms: LR = logistic regression; LASSO = least absolute shrinkage and selection operator (penalized regression); RF = random forest; XGB = extreme gradient boosting (XGBoost); SVM = support vector machine; DL = deep learning; CNN = convolutional neural network; LLM = large language model; NLP = natural language processing; BN = Bayesian network; NB = naïve Bayes; KNN = k-nearest neighbors; Ensemble = combined models (e.g., bagging/boosting/stacking); Other = methods outside the listed families (e.g., generalized linear models beyond LR, Cox/survival models, rule-based systems).*

*Table S 1Full-text exclusions (n = 4)*

| Citation (full) | Record type | Reason for exclusion | Notes |
| --- | --- | --- | --- |
| ClinicalTrials.gov. Enhancing Precise Perioperative Risk Surveillance: Validation of Submaximal Cardiopulmonary Exercise Testing to Usual Care for Detection of Myocardial Injury After Noncardiac Surgery. NCT06474754. 2025. Available at: https://clinicaltrials.gov/study/NCT06474754 | Trial registration A | ClinicalTrials record only; no results / no AI-CDSS data | Trial registration |
| ClinicalTrials.gov. Artificial Intelligence-augmented Perioperative Clinical Decision Support. NCT05284227. 2022. Available at: https://clinicaltrials.gov/study/NCT05284227 | Trial registration B | ClinicalTrials record only; no results / no AI-CDSS data | Trial registration |
| Shen Y, Xie Y, Li L, et al. Risk factors and prediction model of breast cancer-related lymphoedema in a Chinese cancer centre: a prospective cohort study protocol. BMJ Open. 2024;14(12):e089769. doi:10.1136/bmjopen-2024-089769. | Protocol | Protocol only; no results | Protocol |
| Keratoconus: exploring fundamentals and future perspectives — a comprehensive systematic review. *Therapeutic Advances in Ophthalmology*. 2024;16:1–27. doi:10.1177/25158414241232258. | Review lacking AI/PAT focus | Not AI/PAT-relevant or no original AI/ML data | Out of scope (ophthalmology/keratoconus review; not preadmission testing) |

*Full-text reports excluded with reasons (n = 4). Categories reflect protocol/registration status or lack of AI/PAT relevance. No exclusions were made for methodological quality.*

*Table S 2 Title/abstract exclusions (n = 54)*

| **Title** | **First author** | **Year** | **Journal** | **Reason for exclusion** | **DOI** |
| --- | --- | --- | --- | --- | --- |
| Development and validation of a machine learning model for central compartmental lymph node metastasis in solitary papillary thyroid microcarcinoma via ultrasound imaging features and clinical parameters | Han | 2025 | BMC Medical Imaging | Not PAT context (diagnostic/staging radiomics or non-PAT task) | 10.1186/s12880-025-01757-3 |
| MRI-Based Prediction of Meniscal Tear Repairability Demonstrates Limited Accuracy and Reliability | Holland | 2025 | Journal of Clinical Medicine | Not PAT context (diagnostic/staging; orthopedics) | 10.3390/jcm14124160 |
| Prediction of mucinous adenocarcinoma in colorectal cancer with mucinous components detected in preoperative biopsy diagnosis | Ling | 2025 | Abdominal Radiology | Not PAT context (diagnostic/staging radiomics or non-PAT task) | 10.1007/s00261-024-04743-5 |
| Swarm learning network for privacy-preserving and collaborative deep learning assisted diagnosis of fracture: a multi-center diagnostic study | Xie | 2025 | Frontiers in Medicine | Not PAT context (diagnostic/staging radiomics or non-PAT task) | 10.3389/fmed.2025.1534117 |
| AI-driven Characterization of Solid Pulmonary Nodules on CT Imaging for Enhanced Malignancy Prediction in Small-sized Lung Adenocarcinoma | Kudo | 2024 | Clinical Lung Cancer | Not PAT context (diagnostic/staging radiomics or non-PAT task) | 10.1016/j.cllc.2024.04.015 |
| An [18F]FDG PET/3D-ultrashort echo time MRI-based radiomics model established by machine learning facilitates preoperative assessment of lymph node status in non-small cell lung cancer | Meng | 2024 | European Radiology | Not PAT context (diagnostic/staging radiomics or non-PAT task) | 10.1007/s00330-023-09978-2 |
| Preoperative assessment of lymph nodal metastases with [ 68Ga] GaDOTATOC PET radiomics for improved surgical planning in welldifferentiated pancreatic neuroendocrine tumours | Mapelli | 2024 | European Journal of Nuclear Medicine and Molecular Imaging | Not PAT context (diagnostic/staging radiomics or non-PAT task) | 10.1007/s00259-024-06730-w |
| Three-Dimensional Lumbosacral Reconstruction by An Artificial Intelligence-Based Automated MR Image Segmentation for Selecting the Approach of Percutaneous Endoscopic Lumbar Discectomy | Zhu | 2024 | Pain Physician | Not PAT context (diagnostic/staging radiomics or non-PAT task) | PMID: 38324790 |
| “Identifying complication risk factors in reduction mammaplasty: a singlecenter analysis of 1021 patients applying machine learning methods” | Mahrhofer | 2024 | Updates in Surgery | No AI-CDSS (non-AI CDSS or biomarker-only) | 10.1007/s13304-024-01980-7 |
| Machine learning model for preoperative assessment of ultrasound diathermy with implants | Hsu | 2023 | International Journal of Thermal Sciences | Pediatric or non-human — Non-human / lab-only | 10.1016/j.ijthermalsci.2023.108499 |
| Using Machine Learning Methods to Assess Lymphovascular Invasion and Survival in Breast Cancer: Performance of Combining Preoperative Clinical and MRI Characteristics | Xu | 2023 | Journal of Magnetic Resonance Imaging | Not PAT context (diagnostic/staging radiomics or non-PAT task) | 10.1002/jmri.28647 |
| Predictive model for the preoperative assessment and prognostic modeling of lymph node metastasis in endometrial cancer | Asami | 2022 | Scientific Reports | Not PAT context (diagnostic/staging radiomics or non-PAT task) | 10.1038/s41598-022-23252-3 |
| Real-time 3D echocardiographic transilluminated imaging combined with artificially intelligent left atrial appendage measurement for atrial fibrillation interventional procedures | Sun | 2022 | Frontiers in Physiology | Not PAT context (diagnostic/staging radiomics or non-PAT task) | 10.3389/fphys.2022.1043551 |
| Quantification of Intratumoral Heterogeneity Based on Habitat Analysis for Preoperative Assessment of Lymphovascular Invasion in Colorectal Cancer | Su | 2025 | Academic Radiology | Not PAT context (diagnostic/staging radiomics or non-PAT task) | 10.1016/j.acra.2025.03.014 |
| High-risk habitat radiomics model based on ultrasound images for predicting lateral neck lymph node metastasis in differentiated thyroid cancer | Liu | 2025 | BMC Medical Imaging | Not PAT context (diagnostic/staging radiomics or non-PAT task) | 10.1186/s12880-025-01551-1 |
| CTBased Intratumoral and Peritumoral Radiomics Nomograms for the Preoperative Prediction of Spread Through Air Spaces in Clinical Stage IA Nonsmall Cell Lung Cancer | Wang Y | 2024 | Journal of Imaging Informatics in Medicine | Not PAT context (diagnostic/staging radiomics or non-PAT task) | 10.1007/s10278-023-00939-1 |
| Machine learning-based radiomics for guiding lymph node dissection in clinical stage I lung adenocarcinoma: a multicenter retrospective study | Zhang | 2024 | Translational Lung Cancer Research | Not PAT context (diagnostic/staging radiomics or non-PAT task) | 10.21037/tlcr-24-668 |
| Noninvasive Staging of Lymph Node Status in Breast Cancer Using Machine Learning: External Validation and Further Model Development | Hjärtström | 2023 | JMIR Cancer | Not PAT context (diagnostic/staging radiomics or non-PAT task) | 10.2196/46474 |
| Prediction of lymph node status in patients with early-stage cervical cancer based on radiomic features of magnetic resonance imaging (MRI) images | Liu | 2023 | BMC Medical Imaging | Not PAT context (diagnostic/staging; gynecologic oncology) | 10.1186/s12880-023-01059-6 |
| Application of the concept of precision obesity metabolic surgery in laparoscopic Roux-en-Y gastric bypass | Yang | 2022 | Zhonghua wei chang wai ke za zhi = Chinese journal of gastrointestinal surgery | No AI-CDSS (non-AI CDSS or biomarker-only) | 10.3760/cma.j.cn441530-20220717-00317 |
| Assessment of melanoma thickness based on dermoscopy images: an open, web-based, international, diagnostic study | Polesie | 2022 | Journal of the European Academy of Dermatology and Venereology | Not PAT context (diagnostic/staging; dermatology imaging) | 10.1111/jdv.18436 |
| Artificial intelligence-based incisive canal visualization for preventing and detecting post-implant injury, using cone | Jindanil | 2025 | International Journal of Oral and Maxillofacial Surgery | Not PAT context (diagnostic/staging radiomics or non-PAT task) | 10.1016/j.ijom.2025.03.002 |
| A Machine Learning-Based Model for Preoperative Assessment and Malignancy Prediction in Patients with Atypia of Undeterm | Moon | 2024 | Journal of Clinical Medicine | Not PAT context (diagnostic/staging radiomics or non-PAT task) | 10.3390/jcm13247769 |
| Hyperspectral imaging with machine learning for in vivo skin carcinoma margin assessment: a preliminary study | Parasca | 2024 | Physical and Engineering Sciences in Medicine | Not PAT context (diagnostic/staging; dermatology imaging) | 10.1007/s13246-024-01435-8 |
| Prediction of final pathology depending on preoperative myometrial invasion and grade assessment in low-risk endometrial cancer patients: A Korean Gynecologic Oncology Group ancillary study | Jang | 2024 | PLoS ONE | Not PAT context (diagnostic/staging; gynecologic oncology) | 10.1371/journal.pone.0305360 |
| Preoperative assessment of grade, T stage, and lymph node involvement: machine learning-based CT texture analysis in colon cancer | Bülbül | 2024 | Japanese Journal of Radiology | Not PAT context (diagnostic/staging; colorectal oncology) | 10.1007/s11604-023-01502-2 |
| Deep Learning Artificial Intelligence Tool for Automated Radiographic Determination of Posterior Tibial Slope in Patients With ACL Injury | Lu | 2023 | Orthopaedic Journal of Sports Medicine | Not PAT context (diagnostic/staging; orthopedics) | 10.1177/23259671231215820 |
| CT-based deep learning radiomics signature for the preoperative prediction of the muscle-invasive status of bladder cancer | Chen | 2022 | Frontiers in Oncology | Not PAT context (diagnostic/staging; urologic oncology) | 10.3389/fonc.2022.1019749 |
| Outcomes of Artificial Intelligence Volumetric Assessment of Kidneys and Renal Tumors for Preoperative Assessment of Nephron-Sparing Interventions | Houshyar | 2021 | Journal of Endourology | Not PAT context (diagnostic/staging; urologic surgery planning) | 10.1089/end.2020.1125 |
| Machine learning models for prediction of NPVR ≥ 80% with HIFU ablation for uterine fibroids | Yang | 2025 | International Journal of Hyperthermia | Not PAT context (diagnostic/therapeutic response; gynecology) | 10.1080/02656736.2025.2473754 |
| MRI-Based Machine Learning Radiomics for Preoperative Assessment of Human Epidermal Growth Factor Receptor 2 Status in Urothelial Bladder Carcinoma | Yu | 2024 | Journal of Magnetic Resonance Imaging | Not PAT context (diagnostic/staging; urologic oncology) | 10.1002/jmri.29342 |
| Conventional and radiomic features to predict pathology in the preoperative assessment of anterior mediastinal masses | Mayoral | 2023 | Lung Cancer | Not PAT context (diagnostic/staging; thoracic oncology) | 10.1016/j.lungcan.2023.02.014 |
| Multimodal MRI radiomics enhances epilepsy prediction in pediatric low-grade glioma patients | Tang | 2025 | Journal of Neuro-Oncology | Pediatric or non-human — Pediatric cohort | 10.1007/s11060-025-05073-2 |
| Development and validation of a clinic machine-learning nomogram for the prediction of risk stratifications of prostate cancer based on functional subsets of peripheral lymphocyte | Yang | 2023 | Journal of Translational Medicine | Not PAT context (diagnostic/staging; urologic oncology) | 10.1186/s12967-023-04318-w |
| AI-based multimodal prediction of lymph node metastasis and capsular invasion in cT1N0M0 papillary thyroid carcinoma | Peng | 2025 | Front. Endocrinol. | Not PAT context (diagnostic/staging; thyroid oncology) | 10.3389/fendo.2025.1580885 |
| Development and Validation of the Relational Tissue Altered (RTA) Index: Applied Artificial Intelligence for the Assessment of Structural Impact from Laser Vision Correction | Machado | 2025 | Ophthalmology and Therapy | Not PAT context (diagnostic/staging; ophthalmology) | 10.1007/s40123-025-01206-y |
| Development and validation of a machine-learning model for preoperative risk of gastric gastrointestinal stromal tumors. | Liang | 2025 | Journal of gastrointestinal surgery | Not PAT context (diagnostic/staging; surgical oncology) | 10.1016/j.gassur.2024.10.019 |
| Expert-guided StyleGAN2 image generation elevates AI diagnostic accuracy for maxillary sinus lesions | Zeng | 2025 | Communications Medicine | Not PAT context (diagnostic/staging; maxillofacial imaging) | 10.1038/s43856-025-00907-6 |
| Preoperative hemorrhagic risk stratification in pediatric moyamoya disease: a multi-institutional propensity score-matched analysis | Guo | 2025 | Int J Surg | Pediatric or non-human — Pediatric cohort | 10.1097/JS9.0000000000002677 |
| A radiomics-based nomogram for preoperative T staging prediction of rectal cancer | Lin | 2021 | Abdominal Radiology | Not PAT context (diagnostic/staging radiomics or non-PAT task) | 10.1007/s00261-021-03137-1 |
| Quantification of physiological crystalline Lens decentration using swept source OCT | Laubichler | 2025 | European Journal of Ophthalmology | Not PAT context (diagnostic/staging; ophthalmology) | 10.1177/11206721251332759 |
| Radiomic Analysis and Liquid Biopsy in Preoperative CT of NSCLC: An Explorative Experience | Belfiore | 2025 | Thoracic Cancer | Not PAT context (diagnostic/staging; thoracic oncology) | 10.1111/1759-7714.70115 |
| The Performance of Artificial Intelligence in One Anastomosis Gastric Bypass Surgery: Comparative Efficacy of ChatGPT-4.0, ChatGPT-Omni, and Gemini AI | Aksoy E | 2025 | Obesity surgery | Not PAT context (non-PAT task; LLM evaluation) | 10.1007/s11695-025-07794-9 |
| MR radiomics to predict microvascular invasion status and biological process in combined hepatocellular carcinoma-cholangiocarcinoma | Xiao | 2024 | Insights Imaging | Not PAT context (diagnostic/staging; hepatic oncology) | 10.1186/s13244-024-01741-5 |
| Magnetic resonance imaging-based radiomics model for preoperative assessment of risk stratification in endometrial cancer | Wei | 2024 | World J. Clin. Cases | Not PAT context (diagnostic/staging; gynecologic oncology) | 10.12998/wjcc.v12.i26.5908 |
| Preoperative assessment of tertiary lymphoid structures in stage I lung adenocarcinoma using CT radiomics: a multicenter retrospective cohort study | Zhao | 2024 | Cancer Imaging | Not PAT context (diagnostic/staging radiomics) | 10.1186/s40644-024-00813-5 |
| Clinical utility of preoperative pan-immune-inflammation value (PIV) for prognostication in patients with esophageal squamous cell carcinoma | Feng | 2023 | Int. Immunopharmacol. | No AI/CDSS—biomarker-based prognostication; no decision-support model. | 10.1016/j.intimp.2023.110805 |
| Morphometric analysis of dry atlas vertebrae in a northeastern Thai population and possible correlation with sex | Poodendan | 2023 | Surgical and Radiologic Anatomy | Not PAT context (anatomical/morphometry; non-clinical) | 10.1007/s00276-022-03076-6 |
| Multiomics approach for patient stratification and novel target identification in metastatic clear cell renal carcinoma (Meet-URO 31). | Stellato | 2024 | Journal of Clinical Oncology | Trial registration / protocol (no results) | 10.1200/JCO.2024.42.4_suppl.TPS498 |
| Radiomics model based on shear-wave elastography in the assessment of axillary lymph node status in early-stage breast cancer | Jiang | 2022 | Eur. Radiol. | Not PAT context (diagnostic/staging; breast oncology) | 10.1007/s00330-021-08330-w |
| Factors predictive of 90-day mortality after surgical resection for oral cavity cancer: Development of a recursive partitioning analysis for risk stratification | Shinde | 2021 | Head Neck | Not PAT context (postoperative outcomes/risk; head & neck oncology) | 10.1002/hed.26740 |
| Integrating Optimized Multiscale Entropy Model with Machine Learning for the Localization of Epileptogenic Hemisphere in Temporal Lobe Epilepsy Using Resting-State fMRI | Fu | 2021 | Journal of Healthcare Engineering | Not PAT context (diagnostic/localization; neurology) | 10.1155/2021/1834123 |
| Quantitative Prediction of Microsatellite Instability in Colorectal Cancer With Preoperative PET/CT-Based Radiomics | Li | 2021 | Front. Oncol. | Not PAT context (diagnostic/staging; colorectal oncology) | 10.3389/fonc.2021.702055 |
| Contrast-enhanced CT radiomics for preoperative evaluation of microvascular invasion in hepatocellular carcinoma: A two-center study | Zhang | 2020 | Clin. Transl. Med. | Not PAT context (diagnostic/staging; hepatic oncology) | 10.1002/ctm2.111 |

*Title/abstract screening exclusions (total n = 54). Counts derive from Rayyan reviewer decisions after deduplication. Category totals sum to 54.*

*Table S 3 AMSTAR-2 appraisal for reviews*

| **Ref #** | **First author (Year)** | **AMSTAR-2 overall** | **Protocol registered** | **Duplicate selection** | **Comprehensive search** | **ROB methods** | **Meta-analysis methods** | **Publication bias** | **Notes** |
| --- | --- | --- | --- | --- | --- | --- | --- | --- | --- |
| 10 | Buchlak (2020) | Critically low confidence | yes | no | yes | no | NA | NA | No registered protocol; study selection not done independently in duplicate; data extraction in duplicate not reported (≥ 2 critical flaws) |
| 9 | Syversen (2024) | NR | no | no | no | no | NA | NA | Narrative review |
| 11 | Vasileva (2024) | NR | no | no | no | no | NA | NA | Mini-review |
| 27 | Yajima (2025) | Moderate confidence | yes | no | yes | yes | yes | yes | All critical domains met (PROSPERO, duplicate screening/extraction, comprehensive search, QUADAS-2, appropriate meta-analysis & publication bias); two non-critical weaknesses (no itemized list of excluded studies; funding of included studies not systematically reported). |
| 8 | Romito (2025) | NR | no | no | no | no | NA | NA | Narrative review |

*Descriptive AMSTAR-2 appraisal for included reviews (Buchlak 2020; Syversen 2024; Vasileva 2024; Yajima 2025; Romito 2025). NR = not reported; NA = not applicable (e.g., narrative reviews without meta-analysis). AMSTAR-2 overall confidence: High (no critical flaws, ≤ 1 non-critical); Moderate (no critical flaws, > 1 non-critical); Low (1 critical flaw); Critically low (≥ 2 critical flaws). Meta-analysis–specific items are ‘NA’ when no meta-analysis was performed. Appraisals are reported for transparency in a scoping-review context; no reviews were excluded on this basis.*

*Table S 4 PRISMA-ScR checklist with reporting locations*

| **Item** | **Checklist description** | **Where reported (section / locator)** |
| --- | --- | --- |
| Title | Identify the report as a scoping review | Title / Abstract |
| Abstract | Structured summary: objectives, eligibility, sources, charting, results, conclusions | Abstract |
| Rationale | Describe rationale for scoping approach | Intro ¶1–2 'AI-enabled CDSS… relevance for PAT’ |
| Objectives | Explicit statement of objectives/questions | Intro final ¶ 'This review aimed to…' |
| Eligibility criteria | Characteristics of sources (participants, concepts, context) and rationale | Meth — Eligibility Criteria (starts: 'We included studies published between January 1, 2020…') |
| Information sources | All information sources with dates | Meth — Information Sources & Search Strategy (starts: 'A comprehensive search was conducted…') |
| Search | Full electronic search strategy for ≥ 1 database | Supplementary Appendix 1 (database-specific strings) |
| Selection of sources | Process for selecting sources (screening/eligibility) | Meth — Selection Process (starts: 'All identified references were imported into Rayyan…') |
| Data charting process | Methods for charting data and calibration | Meth — Data Charting and Variables (starts: 'We used a predefined extraction schema…') |
| Data items | List and define all variables for which data were sought | Supplementary Table S0; Meth — Data Charting and Variables |
| Critical appraisal | If done, approach for critical appraisal of sources | Methods — Critical Appraisal and Handling of Conference Abstracts (starts: 'Consistent with PRISMA-ScR guidance…') |
| Synthesis of results | Methods of handling and summarizing charted data | Methods — Synthesis of Results (starts: 'We used a narrative synthesis…') |
| Results — Selection | Numbers screened/assessed/included with reasons | Results — Search Results and Study Characteristics; Fig. 1 (114→54→60→56) |
| Results — Characteristics | Characteristics for each included source | Results — General Study Characteristics; Table 1 |
| Results — Critical appraisal | If done, present appraisal of included sources | Supplementary Table S3 (AMSTAR-2) |
| Results — Individual sources | Results of individual sources of evidence | Table 1; domain-specific Results paragraphs |
| Results — Synthesis | Synthesis of findings from charted data | Results — domain synthesis paragraphs |
| Conclusions | Interpretation of results and implications | Discussion |
| Funding | Sources of funding and role of funders | Funding / Acknowledgments |
| Protocol & registration | Availability of protocol/registration | Methods — Registration (OSF DOI 10.17605/OSF.IO/JKCRH; scope amendment dated Aug 23, 2025) |

*PRISMA-ScR items mapped to the reporting locations in this manuscript. Abbreviations:* *PRISMA-ScR = Preferred Reporting Items for Systematic Reviews and Meta-Analyses extension for Scoping Reviews; Intro = Introduction; Meth = Methods; Res = Results.*

*Table S 5 Decision-support function codebook used for synthesis and counting*

| **Function** | **Definition** | **Primary endpoint trigger** | **Examples (from corpus)** | **Edge-case rule** | **Priority rule** |
| --- | --- | --- | --- | --- | --- |
| Risk prediction | Models estimating probability or risk of an adverse outcome or event prior to surgery. | Primary outcome is an event probability or discriminative metric for an outcome (e.g., AUC for PJI, hemorrhage, mortality, delirium). | Maradit Kremers 2025; Chen H 2023; Yu Q 2024. | If later used to route patients but the evaluated endpoint is an outcome probability, classify as risk prediction. | If both risk prediction and triage are plausible, risk prediction takes precedence unless the measured endpoint is operational routing. |
| Triage / test stewardship | Tools designed to route patients, determine test necessity, or allocate venue of care. | Primary endpoint is a triage/test decision or downstream utilization/appropriateness; or the study objective explicitly states routing/appropriateness. | Wongtangman 2023; Woodward 2025. | If the evaluated endpoint is clinical outcome probability rather than routing/appropriateness, classify as risk prediction. | If both apply, classify as triage only when the evaluated endpoint is a triage/test decision or utilization outcome. |
| Workflow optimization | Systems measuring operational efficiency or automation within preadmission processes. | Primary endpoint is time, throughput, automation accuracy, or user preference/usability tied to workflow. | van Giersbergen 2022; Ke 2025. | If only outcome AUCs are reported without operational endpoints, do not assign workflow optimization. | If both workflow and risk are present, assign based on the primary measured endpoint; workflow only if time/efficiency is a reported endpoint. |
| Planning / imaging-driven support | Imaging or radiomics models aimed at staging, planning, or anatomic assessment that inform preoperative decisions. | Primary endpoint is stage/grade, invasion status, anatomic feasibility, or planning metric. | Jiang 2022; Wu Y 2023; Lombaers 2025. | If the endpoint is an outcome probability (e.g., mortality), classify as risk prediction even if imaging is used. | If both staging and outcome are reported, prioritize by the main study objective and abstract language. |

*This codebook defines decision-support functions, the primary endpoint triggers used to assign a study, edge-case rules, and the priority rule applied when a study could plausibly fit more than one function. One primary function was assigned per study for all counts; secondary functions were recorded but not double-counted. Assignment procedure: two reviewers applied this codebook independently with consensus resolution; one primary function per study was used for tallies. Hybrids were flagged in Table 1 but counted once; conference abstracts were synthesized qualitatively only.*
